# The Effectiveness of Different Eradication Schemes for Pediatric *Helicobacter pylori* Infection—A Single-Center Comparative Study from Romania

**DOI:** 10.3390/children9091391

**Published:** 2022-09-14

**Authors:** Oana-Maria Rosu, Nicoleta Gimiga, Gabriela Stefanescu, Ileana Ioniuc, Elena Tataranu, Gheorghe G. Balan, Laura-Mihaela Ion, Doina Anca Plesca, Cristina Gabriela Schiopu, Smaranda Diaconescu

**Affiliations:** 1 Doctoral School, “George Emil Palade” University of Medicine, Pharmacy, Science, and Technology of Targu Mures, 38 Gheorghe Marinescu Str., 540139 Targu Mures, Romania; 2 Faculty of Medicine, “Grigore T. Popa” University of Medicine and Pharmacy, 16 Universitatii Str., 700115 Iasi, Romania; 3 Clinical Department of Pediatric Gastroenterology, “St. Mary” Emergency Children’s Hospital, 62 Vasile Lupu Str., 700309 Iasi, Romania; 4 Gastroenterology and Hepatology Institute, “St. Spiridon” Emergency Hospital, 1–3 Independetei Str., 700115 Iasi, Romania; 5 Clinical Department of Pediatrics, “St. Mary” Emergency Children’s Hospital, 62 Vasile Lupu Str., 700309 Iasi, Romania; 6 Clinical Department of Pediatrics, “Sf. Ioan cel Nou” Emergency Hospital, 720224 Suceava, Romania; 7 Faculty of Medicine and Biological Sciences, “Stefan cel Mare” University of Suceava, 13 University Str., 720229 Suceava, Romania; 8 Faculty of Medicine, “Titu Maiorescu” University of Medicine, 67A Gheorghe Petrascu Str., 031593 Bucharest, Romania; 9 Departament of Pediatrics, Ponderas Academic Hospital, “Regina Maria” Health Network Bucharest, 85A Nicolae G. Caranfil Str., 014142 Bucharest, Romania; 10 Faculty of medicine, “Carol Davila” University of Medicine and Pharmacy, 21 Dionisie Lupu Str., 020021 Bucharest, Romania; 11 Clinical Department of Pediatrics, “Dr. Victor Gomoiu” Clinical Children’s Hospital, 21 Basarabia Str., 22102 Bucharest, Romania

**Keywords:** *Helicobacter pylori*, children, treatment, eradication

## Abstract

Introduction: In Romania, studies on the pediatric population regarding *H. pylori* infection or bacterial resistance to antibiotics are limited. Eradication treatment of this infection still raises important problems in medical practice. This study aims to compare the effectiveness of three eradication therapies used against *H. pylori* infection in the pediatric population. Methods: The prospective study enrolled children aged 6–17 years who were first diagnosed with *H. pylori* infection. Patients received a randomized treatment either the therapy with clarithromycin (CLR), the therapy with metronidazole (MTZ) or sequential therapy. The effectiveness of the eradication treatment was evaluated after 4–8 weeks from the end of the therapy by testing fecal antigen. Results: 149 patients were enrolled over 18 months. The eradication rates were 49.5% for the treatment scheme with proton pump inhibitor (PPI) + amoxicillin (AMO) + MTZ, 26.7% for PPI + AMO + CLR and 23.8% for sequential therapy. MTZ therapy was superior to CLR therapy, but sequential therapy was not. Side effects were recorded for PPI + AMO + CLR with 39.6%, followed by sequential therapy 37.7%, and only 22.6% for PPI + AMO + MTZ. Conclusions: Therapy with MTZ can achieve a higher eradication rate as a first-line treatment in the case of *H. pylori* infection. Taking into account that Romania is in an area with increased resistance to CLR, MTZ therapy could be a promising alternative.

## 1. Introduction

*Helicobacter pylori* (*H. pylori*) causes one of the most frequent infections, globally affecting over 4 billion people which is transmitted within the family and is most often acquired in childhood [1,2]. This opportunistic human pathogen reaches the stomach by any possible route (e.g., oral-oral, fecal-oral, contaminated water and food, etc.), and without treatment it can persist throughout life [2,3]. Moreover, infection with *H. pylori* can be acquired from parents or siblings [4]. Rowland et al. have demonstrated that having an infected family member (mother or older brother) or using a bottle after the age of 24 months are risk factors for *H. pylori* infection [5]. Cervantes et al. suggest through their study that transmission occurs from older siblings to younger ones [6]. Moreover, there is the possibility that the infection of the younger brother acquired from the older brother, may be more persistent. The infection of the older brother precedes the infection of the younger brother, being an important source of *H. pylori* transmission, especially when the brothers are close in age [6]. Konno et al. found that there is a predominance of transmission of *H. pylori* infection from mother to child. The mother spends more time in contact with the child than the father. Most often, the mother takes care of the practices for raising and caring for children, including breastfeeding, bottle feeding or using a pacifier [7].

The prevalence of *H. pylori* infection increases inversely and proportionally with the socio-economic level. In Romania, the few studies on the prevalence of *H. pylori* infection among children report a prevalence of 40% in 2002 and 25% in 2018 [8,9].

According to the European Society of Pediatric Gastroenterology and Nutrition (ESPGHAN)/North American Society of Pediatric Gastroenterology and Nutrition (NASPGHAN) guidelines, the first-line treatment should be prescribed according to the antimicrobial susceptibility of *H. pylori*, and when it cannot be determined, the therapeutic scheme should be adapted according to the regional tendency of antimicrobial resistance [10]. The treatment of *H. pylori* infection involves a triple therapy with a PPI along with AMO and another antibiotic, such as CLR or MTZ [10]. In areas with a low resistance to CLR, treatment can be empirical and contain CLR according to current recommendations. If eradication therapy with PPI, AMO and CLA has failed, PPI, AMO and MTZ can be prescribed without additional susceptibility testing. The ideal treatment administration period is 14 days, but 10 days can be accepted if local efficiency is proven. Due to the low possibilities of determining bacterial resistance to antibiotics, in clinical practice, the treatment could be based on the patient’s history of previous exposure to antibiotics and local antibiotic resistance, but there is not enough data on the effectiveness of eradication regarding the most used antibiotics [10].

Anti-*H. pylori* therapy is a current problem due to the change in sensitivity of the bacteria to antibiotics. This may be due to the frequent and uncontrolled use of antibiotics to treat various pediatric respiratory or otorhinolaryngology infections. Unfortunately, Romania maintains a leading position in the European Union regarding the use of antibiotics without medical prescription, with an estimate of 30% [11,12].

Savoldi et al. performed a systematic review and meta-analysis to determine the distribution of *H. pylori* resistance to the most widely used antibiotics in the regions of the World Health Organization [13]. Since there are no clear data on the global distribution of resistance or clinical effects, the authors summarized data from the literature. They determined a prevalence of antibiotic resistance by age group. Thus, in the case of children from the American regions there is a prevalence of 19% for CLR and 22% for MTZ, in the Southeast Asian region 16% for CLR and 59% for MTZ, and in the European region 24% for CLR and 20% for MTZ [13]. Boyanova et al. have carried out in a Bulgarian study, along with researching data from the literature, an analysis of the rate of multiple antibiotic resistance of *H. pylori* strains. The determined resistance rates were 7.5% for AMO, 25.5% for MTZ and 34.0% for CLR. The combined resistance rate was 19.8%, including double, triple and quadruple resistance in 13.2% (14 strains), 5.7% (6) and 0.9% (1) of the strains, respectively [14].

Statistical data from a meta-analysis carried out in Europe on adults show resistance of 18%, 32% and 11% for CLR, MTZ and levofloxacin. In the case of children, in Europe, the use of antibiotics on a large scale determined resistance rates between 0.8% (The Netherlands) and 49.2% (Spain) [1,15]. A study carried out on adults in the north-east of Romania determined a resistance of 20.3%, but currently there are no determinations of the resistance rate of *H. pylori* to antibiotics in the pediatric population in Romania [16].

In Romania, there are few studies on gastric infection with *H. pylori* among children and even fewer on the effectiveness of their treatment [17,18]. This study aims to compare the effectiveness of the therapeutic schemes with PPI + AMO + MTZ for 14 days, PPI + AMO + CLR for 14 days and sequential therapy for 10 days.

## 2. Materials and Methods

The prospective study was carried out in the Pediatric Gastroenterology unit of St. Mary Children’s Hospital in Iasi, Romania, over 18 months (2019–2020) and has the approval of the Ethics and Research Committee of the hospital, registered with no. 4363/20.02.2019.

The study included 149 patients aged between 6 and 17, who performed upper digestive endoscopy for various gastroenterological conditions. The diagnosis of *H. pylori* infection was determined by histopathology and rapid urease test. The informed consent was signed by the parents of the children included in the present study.

Exclusion criteria included: patients under 6 years old and over 18 years, those who received PPI treatment, antibiotics or antibacterial treatment four weeks before the procedure, and patients with associated pathologies, such as Celiac Disease or Crohn’s Disease.

According to ESPGHAN guidelines, the prescribed treatment was based on body weight. Due to unknown resistance of *H. pylori* to antibiotics and because the determination of antibiotic resistance was not possible, eradication regimens were randomly assigned to patients. The patients were divided into three groups, each group having a different therapeutic scheme (described in Table 1). Three therapeutic regimens were used, and for each of them were recorded side effects, compliance with the treatment received and the highest rate of eradication.

After finishing the treatment, the patients enrolled in the study completed a questionnaire. The questionnaire contained data related to duration of treatment, a reason to stop it, (if it were the case), side effects related to therapy. The ESPGHAN guidelines recommend that monitoring of the effectiveness of eradication therapy should be performed within 4 to 8 weeks after the end of antibiotic therapy. The guideline state that non-invasive tests such as antigen testing fecal or 13C-UBT should be used to confirm eradication of infection [10]. Our study used methods to monitor the eradication of *H. pylori* infection by performing a fecal antigen after 4–8 weeks or more from the end of the treatment.

Statistical analysis was performed using IBM SPSS Statistics for Windows, v20.0 (Armonk, NY, USA). Percentages, standard deviations and averages were used for descriptive data analysis, and the Chi-square test and Pearson correlations for quantitative data analysis (*p* < 0.05 was considered statistically significant).

## 3. Results

Out of 149 patients enrolled in study, 103 were girls (69.12%) and 46 were boys (30.87%). The average age of the patients was 13.20 ± 3.30. The most common symptom for performing upper digestive endoscopy was epigastric pain (56.37%), followed by recurrent abdominal pain (16.10%) or epigastric pain in association with other symptoms (15.43%). All of the patients in the study were at the first diagnosis and the first treatment against *H. pylori* infection. The characteristics of the patients enrolled in the study, the indications for diagnosis and the prescribed treatment are shown in Table 1.

The prescribed eradication therapies were PPI + AMO + MTZ for 14 days (41.62%), PPI + AMO + CLR for 14 days (32.88%) and sequential therapy with PPI + AMO for 5 days followed by another 5 days with PPI + CLR + MTZ (25.50%). The eradication treatment and prescribed doses were according to the current ESPGHAN guidelines (Table 2).

Treatment compliance was slightly higher among girls (85/103, 82.52%) compared to boys (36/46, 78.26%). The general eradication rate of the patients studied was 67.79% (101/149), but the eradication of *H. pylori* infection failed for 32.21% (48/149). The efficiency of PPI + AMO + MTZ therapy was 80.64% (50/62), PPI + AMO + CLR therapy was 55.11% (27/49) and sequential therapy was 63.15% (24/38), as shown in Table 3.

Side effects to treatment were experienced by 35.57% of patients (53/149), of which 39.6% (21/49) were those who received PPI + AMO + CLR, followed by those who received sequential therapy 37.7% (20/38) and 22.6% (12/62) of those who received PPI + AMO + MTZ (*p* = 0.001) (Table 4).

Among the types of side effects experienced by the patients who stopped the treatment, the most frequently declared were nausea and epigastric pain (17.9%), followed by nausea and vomiting (14.3%), and 10.7% had associations between nausea, epigastric pain, headache and/or vomiting. Most side effects disappeared once the treatment was stopped. Moreover, 21.4% stopped the treatment without having side effects (*p* = 0.000).

A total of 41.5% (22/53) stopped the treatment due to side effects, and 6.3% stopped the treatment of other reasons (Table 5).

## 4. Discussion

Eradication therapy for *H. pylori* infection is recommended, according to current ESPGHAN guidelines, when there are endoscopic changes and at least one positive test for *H. pylori*. The diagnosis of *H. pylori* infection from our study was made following the upper digestive endoscopy with gastric biopsy and histopathology. The most common endoscopic aspects were hyperemic lesions and nodular lesions of the gastric mucosa. The recommended antimicrobial treatment is combination therapy with PPIs and at least two antibiotics [10]. Dissimilar to eradication schemes for adults, there are a limited number of antibiotics that can be administered to children. Children have a much lower tolerance to the side effects of antibiotics than adults, but also a higher rate of subsequent bacterial reinfection [17]. Due to all these factors, the treatment of *H. pylori* infection in the pediatric population is quite limited, so it can represent a challenge in clinical practice. In our study, three therapeutic regimens were used according to the updated international guidelines regarding the eradication of infection among the pediatric population, combining PPI with AMO and CLR or MTZ. There are no studies in Romania that investigate other treatment for the eradication of pediatric *H. pylori* infection apart from the standard triple therapy with CLR.

In general, the failure of eradication therapy is associated with several factors, such as low compliance, bacterial resistance or incorrect treatment [17]. However, non-adherence and non-compliance with treatment remain one of the major factors of failure of the eradication scheme, which can later determine bacterial resistance to antibiotics. Treatment non-compliance can have several causes, but an important role is played by the side effects associated with eradication therapy. Several studies claim the fact that antibiotic intolerance plays a huge role in therapy failure [19].

In our study, the number of infected girls is higher than boys (69.12% vs. 30.87%). We presume that this resulted from a higher number of admissions in girls compared to boys. At the same time, the main reason for presentation for all our patients was epigastric pain. According to other authors, girls seem to have a lower pain tolerance threshold than boys and there are studies regarding the different tolerance between girls and boys towards pain [20]. Kim et al. conducted a review demonstrating that the clinical presentation in women and men may differ due to gender differences represented by a variety of physiological and psychological factors [21]. Similarly, Casale et al. believes that gender differences in pain are conditioned by anatomical, neural, hormonal, social and cultural factors [22]. The review by Bartley et al. summarizes clinical and epidemiological findings, concluding that women are at increased risk for chronic pain and may experience more severe clinical pain [23]. A study by Hafeez et al. report that, although there was better treatment compliance among boys, there was no significant association between gender and stopping the medication [24]. On the contrary, in our study, a better compliance can be observed among girls (85.2%) than among boys (78.26%), but similarly, without a statistically significant correlation between compliance and gender (*p* = 0.558) or age (*p* = 0.060) of the patients.

Very few antibiotics can be used for the eradication scheme of pediatric *H. pylori* infection, and the increase in bacterial resistance becomes a significant problem in clinical medical practice. Liou et al. demonstrate through a study on adults that empiric therapy based on antibiotic exposure history for other common infections can be as effective as genotypic resistance-guided therapy [25]. To eradicate *H. pylori* infection among children, the recommended antibiotics are AMO, CLR and MTZ. The current ESPGHAN/NASPGHAN (2016) and JSPGHAN (2020) guidelines recommend PPI + AMO + CLR therapy for 14 days if antimicrobial susceptibility is unknown [26]. At present, CLR and MTZ present bacterial resistance at a global level, which causes a decrease in the rate of bacterial eradication among the pediatric population [17]. As an alternative therapy to the first-line therapy for *H. pylori* infection among children, the regional resistance of the bacteria to antibiotics, especially to CLR and MTZ, should be taken into account. Lai et al. incorporate several statistics in which resistance has increased since 2012, CLR 11.9%, MTZ 10.1%, AMO 0.6%, until 2018 in some regions of the World Health Organization by 15%, considering that regional resistance must be monitored longitudinally, especially in areas with low eradication rates [26]. Meta-analyses carried out on adults claim that a longer duration of treatment increases the eradication rate, but an increased duration of treatment is associated with an increased incidence of side effects. The current ESPGHAN guidelines admits that there are few well-conducted studies in the pediatric population regardless of the duration or type of treatment, the eradication rates of *H. pylori* infection do not reach the 90% recommended per protocol. Although considered optimal therapy against *H. pylori* when it reaches 90%, such values are rarely proven in current practice [10]. In our study, the eradication efficacy for the PPI + AMO + MTZ treatment protocol was 80.64%, which is statistically significant (*p* = 0.001). Although far below the recommended eradication rate, our results seem to be close to other studies. In the meta-analysis performed by Lai et al. regarding the treatment of pediatric *H. pylori* infection, it was found that the eradication rates with the standard triple therapy with CLR administered for 14 days are 54.2–74.1%, and for the combination of PPI + AMO + MTZ for 10 days it reaches a rate of 76.7%. In the same meta-analysis, sequential therapy for 10 days indicates an eradication rate of 80.4–91.2%, except for one study that shows that with a 14-day administration, the eradication rate is 69.5%. However, the eradication rates are not sufficiently satisfactory related to the administered scheme, and it concludes that the studies show contrary results [26]. In a study by Zhou et al., the eradication rate with standard triple therapy with CLR was only 60% [27]. In our study, the eradication rate for PPI + AMO + CLR therapy was lower than that, 55.11%, but similar to sequential therapy by 63.15%. Sequential therapy has the disadvantage of exposing the child to three antibiotics, although the eradication rate is better than that of triple therapy with CLR.

The side effects of eradication therapy were mentioned in some studies which, similarly to our study, disappeared after stopping the treatment [26]. In the meta-analysis by Fischbach et al., as well as in the study by Hafeez et al. 50% of patients had side effects to triple therapy. In a study conducted in China, the incidence of side effects was 12.3%, with no statistic semnification difference between the therapeutic regimens [27]. Hafeez et al. report that side effects nausea, vomiting, diarrhea and abdominal pain were declared by 74% of the patients who received the treatment scheme with CLR and by 55% of those who received the scheme with MTZ [24]. In another study conducted by Bontems et al., in which he compared PPI + AMO + CLR with sequential therapy, the side effects were mainly abdominal pain 20%, diarrhea 14%, nausea 6% and vomiting 2% [28]. In our study, the index of side effects in enrolled patients had a percentage of 35.57%, being statistically significant (*p* = 0.001). Similar percentages were for PPI + AMO + CLR and sequential therapy, 39.6% and 37.7%, respectively, the lowest incidence of side effects being 22.6% for the PPI + AMO + MTZ scheme. Depending on the administered therapeutic scheme, the incidence percentages of side effects differ. The highest percentage, of 52.63%, was in the case of sequential therapy, followed by PPI + AMO + CLR 42.76%, the lowest percentage being in the case of PPI + AMO + MTZ therapy 19.35%. As the main symptoms of the side effects were vomiting, nausea, diarrhea and epigastric pain in the case of the PPI + AMO + CLR, vomiting, nausea and epigastric pain for the sequential therapy, and vomiting and nausea for the PPI + AMO + MTZ. However, the symptomatology determined by the side effects to the treatment does not correlate statistically significantly (*p* = 0.515). Moreover, there is a statistically insignificant correlation (*p* = 0.432) between side effects and discontinuation of treatment, and a statistically insignificant correlation between the presence of side effects and age (*p* = 0.062).

However, effective communication between doctor, patient and parent/s is necessary to be able to explain in detail the therapeutic scheme, the existence of common side effects and the importance of treatment compliance, all of which are essential for a successful *H. pylori* eradication therapy.

Our study has several limitations. We did not have the possibility, at the time of the study, to determine microbial resistance in any of the patients studied. Our study was partially carried out during the COVID-19 pandemic, and for this reason we lost patients from the study because they did not return for follow-up. Another limitation is related to the fact that the study does not address the general population and is carried out in a single hospital. Furthermore, due to the combination of antibiotics in the eradication schemes, we cannot correlate the side effects with a specific drug. In addition, it did not record data about the commercial form or the preparation used exclusively by the patients enrolled in the study. However, our study presents recent data from clinical practice and the prescribed treatments were in accordance with updated international guidelines. To the best of our knowledge, this is the first study in our country that compares the efficiency of various therapeutic regimens in children.

## 5. Conclusions

Since Romania is still in an area with unknown resistance of *H. pylori* in the pediatric population, for a good eradication rate and the lowest possible incidence of side effects, it is necessary to explore therapeutic regimens in order to determine the most effective treatment schemes. Triple therapy with PPI, AMO and MTZ can achieve a higher eradication rate as a first-line treatment in the case of *H. pylori* infection. This study is the only one of its kind in Romania regarding the effectiveness of a therapy scheme for the eradication of pediatric *H. pylori* infection. Moreover, the side effects to the treatment can be the cause of the interruption of the treatment and the failure of the therapeutic scheme.

## Figures and Tables

**Table 1:** The characteristics of the patients enrolled in the study.

Characteristics	Variables	Number of Patients	%
Sex	Female	103	69.12
	Male	46	30.87
Age	6–9 years	26	17.46
	10–14 years	53	35.57
	15–17 years	70	46.97
Indications for upper digestive endoscopy and *H. pylori* testing	Epigastric pain	84	56.37
	Recurrent abdominal pain	24	16.10
	Epigastric pain and other symptoms	23	15.43
	Recurrent abdominal pain and other symptoms	6	4.04
	Dyspepsia	2	1.34
	Other symptoms	10	6.72
Prescribed treatment	PPI + AMO + MTZ 14 days	62	41.62
	PPI + AMO + CLR 14 days	49	32.88
	PPI + AMO 5 days followed by PPI + CLR+MTZ 5 days	38	25.50

PPI—proton pomp inhibitor; AMO—amoxicillin; CLR—clarithromycin; MTZ—metronidazole.

**Table 2:** Standard PPI and antibiotic prescription doses for body weight.

Drug	Range for Bodyweight	Doses
PPI	15–24 kg	20 mg + 20 mg
	25–34 kg	30 mg + 30 mg
	>35 kg	40 mg + 40 mg
Amoxicillin	15–24 kg	500 mg + 500 mg
	25–34 kg	750 mg + 500 mg
	>35 kg	1000 mg + 1000 mg
Metronidazole	15–24 kg	250 mg + 250 mg
	25–34 kg	500 mg + 250 mg
	>35 kg	500 mg + 500 mg
Clarithromycin	15–24 kg	250 mg + 250 mg
	25–34 kg	500 mg + 250 mg
	>35 kg	500 mg + 500 mg

Recommended doses for morning and evening.

**Table 3:** The effectiveness of the prescribed treatment.

Treatment Regimen	Fecal Antigen (−)		Fecal Antigen (+)		*p*
	*n*	%	*n*	%	
PPI + AMO + MTZ 14 days	50	49.5	12	25.0	0.001
PPI + AMO + CLR 14 days	27	26.7	22	45.8	
PPI + AMO 5 days followed by PPI + CLR + MTZ 5 days	24	23.8	14	29.2	

Proportion analyses were performed with Pearson Chi-Square Tests.

**Table 4:** Side effects depending on the therapeutic scheme.

Treatment Regimen	Side Effects				*p*
	Yes		No		
	*n*	%	*n*	%	
PPI + AMO + MTZ 14 days	12	22.6	50	52.1	0.001
PPI + AMO + CLR 14 days	21	39.6	28	29.2	
PPI + AMO 5 days followed by PPI + CLR + MTZ 5 days	20	37.7	18	18.8	

Proportion analyses were performed with Pearson Chi-Square Tests.

**Table 5:** Correlation between side effects and interruption of treatment.

Side Effects	Interruption of Treatment				
	Yes		No		*p*
	*n*	%	*n*	%	
Yes	22	41.5	6	6.3	0.432
No	31	58.5	90	93.8	

Proportion analyses were performed with Pearson Chi-Square Tests.

## Data Availability

Not applicable.
